# Short‐distance barriers affect genetic variability of *Rhizophora mangle* L. in the Yucatan Peninsula

**DOI:** 10.1002/ece3.4575

**Published:** 2018-10-19

**Authors:** Diana J. Cisneros‐de la Cruz, Jaime Martínez‐Castillo, Jorge Herrera‐Silveira, Laura Yáñez‐Espinosa, Matilde Ortiz‐García, Roberth Us‐Santamaria, José L. Andrade

**Affiliations:** ^1^ Centro de Investigación Científica de Yucatán (CICY) Mérida México; ^2^ Centro de Investigación y Estudios Avanzados (CINVESTAV) Mérida Mérida Mexico; ^3^ Instituto de Investigaciones de Zonas Desérticas Universidad Autónoma de San Luis Potosí (UASLP) San Luis Potosí Mexico

**Keywords:** ecological barriers, microsatellite, phosphorous, salinity, scrub mangrove, Yucatan

## Abstract

The environmental variability at local scale results in different physiognomic types of mangrove forest. However, this variability has never been considered in studies of mangrove genetic variability. This study analyzed the genetic and morphological variability and structure of *Rhizophora mangle* at regional and local scales in the Yucatan Peninsula. Thirteen mangrove populations (eight scrub and five tall), located in seven sites, were sampled, and their morphological variability and relationship with the availability of phosphorus and salinity were analyzed. The diversity and genetic structure were estimated at different hierarchical levels with nine microsatellites, also Bayesian inference and Principal Coordinates Analysis were used. We found a great morphological variability of *R. mangle* that responded to local environmental variability and not to the precipitation gradient of the peninsula. The genetic diversity found in the peninsula was greater than that reported for other populations in Mexico and was grouped into two regions: the Gulf of Mexico and the Caribbean Sea. At a local scale, tall and scrub mangroves had significant genetic differentiation suggesting that ecological barriers promote genetic differentiation within sites. These results need to be considered in future population genetic studies and for mangrove management and conservation.

## 1 INTRODUCTION

Identifying the evolutionary processes controlling genetic structure and morphological diversity is a central question of evolutionary biology (Dewoody, Trewin, & Taylor, 2015). While the genetic structure of populations is typically driven by gene flow, genetic drift and mutation of novel alleles (Slatkin, 1993; Wright, 1931), phenotypic differentiation among populations could reflect a balance between natural selection in the local environment, migration of alleles via gene flow, and, at a lower frequency, the acquisition of novel traits through mutation (Dewoody et al., 2015; Nosil & Crespi, 2004). Therefore, the combined use of genetic, morphological, and environmental characteristics may help to understand the patterns of variation and differentiation of a species comprehensively (Bruschi, Angeletti, González, Signorini, & Bagnoli, 2013; Orsini, Vanoverbeke, Swillen, Mergeay, & Meester, 2013).

Studies on the genetic and morphological variability of a species population, at local and regional scale, would allow us to understand the potential response of this species to environmental changes. Also, these studies can help us to understand the mechanisms that regulate the population´s structure and the ecological processes behind it, as well as their role in the ecosystem services offered (Salgado‐Negret & Paz, 2016). Furthermore, the success of restoration, management, and conservation projects depends on conserving and prioritizing the greater genetic and functional variability (Richards, Wares, & Mackie, 2010). However, studies that correlate morphological and genetic differences for the same species geographically separated are very scarce. Recently, conceptual frameworks have identified local genetic adaptation as an important driver of population genetic structure, but only some studies focus on tree species, and a few focus on mangroves (Farnsworth, 1998; Nosil, Funk, & Ortiz‐Barrientos, 2009; Orsini et al., 2013).

Mangroves are intertidal ecosystems located in the tropical and subtropical coasts of the world (Tomlinson, 1986). These forests have a great ecological and economic importance as they shelter a supreme diversity of fauna and provide important ecosystem services (Alongi, 2008). The structure and floristic compositions of mangroves change according to geographical and latitudinal gradients (Lot, Vázquez‐Yanez, & Méndez, 1975; Lugo & Snedaker, 1974). At the local level, their composition, structure, and function vary according to environmental gradients, physiological preference, and flood tolerance of the species (McKee, 1996; Rabinowitz, 1978), which result in different type of physiognomic mangrove forests. Lugo and Snedaker (1974) describe five main types: riverine, basin, fringe, overwash, and scrub. This classification scheme is a supplement to the classical zonation patterns described for mangroves in different parts of the world, which has had a ubiquitous success and strengthens the importance of the distribution of mangrove species and physiognomic units (Lugo & Snedaker, 1974).

The scrub mangrove is characterized by dense, low‐height (<3 m) and generally monospecific forests of *Avicennia germinans* [(L.) Stearn] or *Rhizophora mangle* L. (Lugo & Snedaker, 1974; Trejo‐Torres, Duran, & Olmsted, 1993). This type of mangrove has been the focus of several studies for its contrasting characteristics compared to tall mangrove trees (Cheeseman & Lovelock, 2004; Lin & Stenberg, 1992a; Naidoo, 2010; Yáñez‐Espinosa & Flores, 2011); its short stature has mainly been attributed to limitation of nutrients, in particular phosphorus (Feller, McKee, Whigham, & O'Neill, 2003; Lovelock, Feller, Ball, Engelbrecht, & Ewe, 2006; Lovelock, Feller, McKee, Engelbrechts, & Ball, 2004) and hypersalinity or salinity fluctuations that reduce water availability, photosynthesis rate, and tree growth (Hao et al., 2009; Lin & Stenberg, 1992b; Naidoo, 2006). Indeed, one study suggests that the short stature of this type of mangrove could be a genetic expression rather than a phenotypic expression (Lara‐Domínguez et al., 2005).

The genetic variability of mangroves species, particularly that of *R. mangle*, began to be studied with the use of biochemical markers by Dodd, Rafii, Fromard, and Blasco (1998), and in the past decade with DNA molecular markers by Arbeláez‐Cortes, Castillo‐Cárdenas, Toro‐Perea, and Cárdenas‐Henao (2007). Recently, several studies (the majority of them using microsatellite molecular markers) have reported a great genetic variability within populations of *R. mangle* in America (Albrecht, Kneeland, Lindroth, & Foster, 2013; Arbeláez‐Cortes et al., 2007; Bruschi et al., 2013; Cerón‐Souza et al., 2015; Cerón‐Souza, Bermingham, McMillan, & Jones, 2012; Pil et al., 2011). However, none of these studies have considered the physiognomic type of mangrove in their sampling plan. In the Yucatan Peninsula, *R. mangle* occurs in forests of fringe, basin, “petén” (associated with freshwater inputs) and scrub, which is also the most extensive mangrove type (Adame et al., 2013; Herrera‐Silveira et al., 2016; Zaldívar‐Jiménez et al., 2010). Moreover, although this region represents more than half of the extension of mangrove in Mexico, no studies of genetic variability of *R. mangle* have been carried out (Rodríguez‐Zúñiga et al., 2013; Spalding, Kainuma, & Collins, 2010).

Accordingly, the principal aim of this work was to evaluate the morphological and genetic structure and variability of *R. mangle* at regional and local scales in the Yucatan Peninsula, Mexico. In addition, we examined the relation of morphological variability to salinity and phosphorous availability. The following specific questions were addressed which are as follows: (a) How the morphological variability of *R. mangle* relates to salinity and phosphorus availability in the Yucatan Peninsula? (b) what are the levels of diversity and genetic structure in the Yucatan Peninsula?, and (c) at local scale, do the two physiognomic types of *R. mangle,* tall and scrub, affect the genetic structure and diversity within a site? We would expect that considering the influence of salinity and phosphorus on mangrove morphology, the sites with the highest salinity and lowest phosphorus availability will correspond to scrub populations. Also, the tall and scrub *R. mangle* will show genetic differentiation within a site.

## 2 MATERIALS AND METHODS

### 2.1 Study area and populations sampled

This study was made along the northern and eastern coasts of the Yucatan Peninsula, which is a low elevation karstic platform (slope <1%) with irregular topography (Pannier, 1992). The climate is generally warm and humid and is characterized by three seasons: dry (March–May), rainy (June–October), and early‐dry (locally named “nortes”; November‐February) (Zaldívar‐Jiménez et al., 2010).

**Figure 1:**
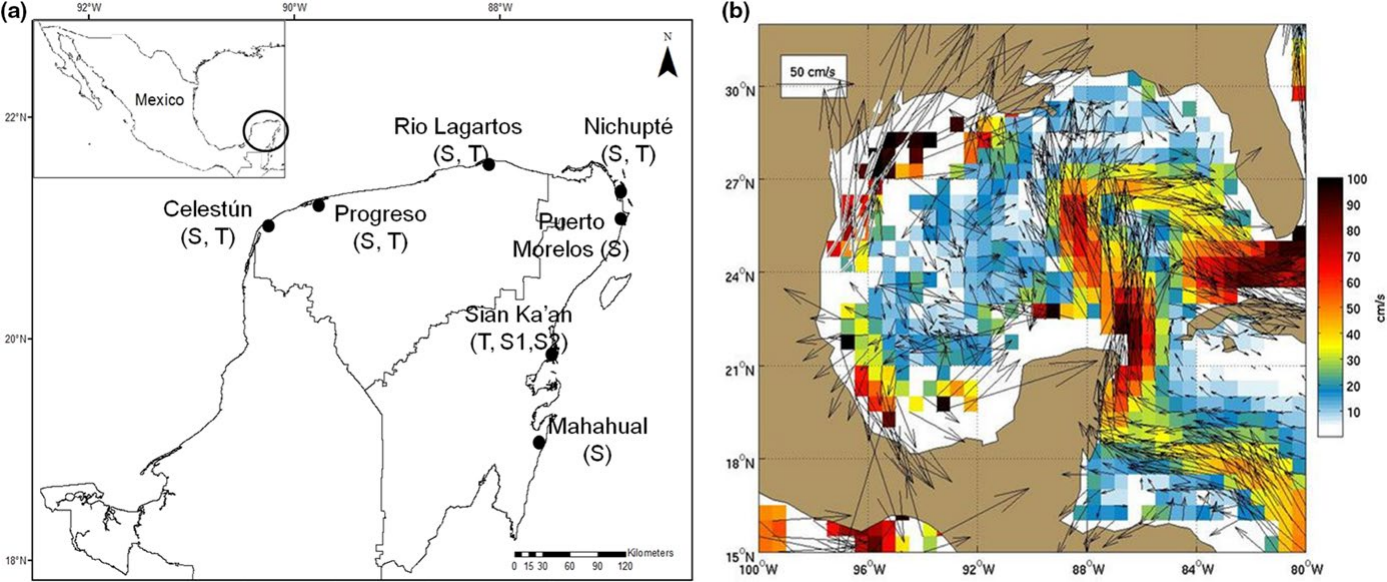
(a) Sampled sites for *Rhizophora mangle* populations in the Yucatan Peninsula, the physiognomic types are indicated by (*T*) for tall and (*S*) for scrub. (b) Ocean currents of the Yucatan Peninsula (Laurindo et al., 2017)

Seven sites with the presence of *Rhizophora mangle* (Rhizophoraceae) forests along the coast of the Yucatan Peninsula were selected (Figure 1a); in each site, sampling points were chosen according to the physiognomic type of mangrove, previously designed in the Mexico Mangrove Monitoring System (Herrera‐Silveira et al., [Link]). All sites but one (Progreso) are priority international wetlands (CONANP (Comisión Nacional de Áreas Naturales Protegidas), 2014). In total, thirteen points were sampled; each one was considered a different population and classified, according to their morphological structure, as tall (>3 m) or scrub (<3 m) (Figure 1a).

### 2.2 Morphological and environmental characterization

In each population, ten adult individuals (each with at least 10 m distance among each other) were randomly selected. We measured the following: (a) leaf length and width (cm; 3 leaves per individual; the third leaf from the leaf primordium was always selected); (b) length of ten propagules with a graduated ruler (cm); (c) height (m), measured with a Vertex Laser (VL402, Haglöf, Sweden); and (d) stem diameter (cm; performed 20 cm above the last root with a diameter tape or a vernier for the scrub mangrove). At each site, interstitial salinity (~30 cm depth) was measured with an YSI Pro 2030 portable conductivity meter (YSI, Yellow Springs, USA). Also, a soil core with a depth of 30 cm was obtained and transported to the laboratory to measure total phosphorus (P) and extractable P. Total P was determined as orthophosphate after Aspila, Agemian, and Chau (1976) and Parson, Maita, and Lalli (1984). Extractable P was analyzed after Olsen (1954).

#### 2.2.1 Data analysis

A Canonical Discriminant Analysis was performed to know the morphological differences between populations of Yucatan Peninsula and the most discriminant variables. Also, to distinguish a pattern among the environmental variables to the mangrove type, a Principal Component Analysis was used. Finally, a Canonical Correlation Analysis was applied to identify the correlation among the morphological variables: height, length of the propagule and to include the foliar characters, leaf area was calculated considering the leaf as an oval (Af = π × *r*_1_ × *r*_2_; *r*_1_: leaf width, *r*_2_: leaf length); and the environmental variables: salinity and extractable P. Analyzes were realized in XLSTAT v.7.5.2. (XLSTAT, Addinsoft, USA, 2007).

### 2.3 Molecular characterization

For the molecular analysis, leaf samples (young leaves without apparent damage) from 130 individuals of *R. mangle* were collected from August 2014 to March 2015. Ten individuals (separated by at least 20 m distance from each other to avoid kinship) per population were chosen for leaf sampling. Polyvinylpyrrolidone (PVP) was applied to each leaf in the abscission zone to avoid oxidation; leaf samples were stored at −20°C for further analysis.

DNA was isolated by the modified CTAB/PVP method (Reyes‐Medina, 2012). Previously, tissue was macerated in liquid nitrogen, resuspended in 1,000 μl of 2% CTAB plus 10 μl of β‐mercaptoethanol, and then incubated at 65°C for 3 hr. Samples were allowed to cool and 30 μl of 20 μg/μl RNAse was then added and incubated at 37°C for 30 min; the enzyme was inactivated at 65°C for 10 min. Thereafter, it was centrifuged at 18,000 *g* for 20 min, 700 μl of the supernatant were transferred and extracted with 650 μl of 24:1 (v/v) chloroform‐isoamyl alcohol, and centrifuged at 18,000 *g* for 10 min to separate the phases. This process was repeated twice. The resulting supernatant DNA was precipitated with 700 μl of cold isopropanol and 35 μl of 3 M sodium acetate, left overnight incubating at −20°C and centrifuged for 20 min at 18,000 *g*. The pellet was washed with 200 μl of cold absolute ethanol and centrifuged for 6 min at 18,000 *g*. The DNA obtained was resuspended with 70 μl of 1X TE and stored at −20°C for further analysis. The quality of the extracted DNA was verified by electrophoresis in 1% agarose gels stained with ethidium bromide (10 mg/ml). The DNA was quantified on a NanoDrop 2000 UV‐Vis spectrophotometer (Thermo Scientific), and subsequently, the concentration of each sample was homogenized to 25 μg/μl and stored at −20°C until further use.

For the microsatellite technique, nine loci reported as polymorphic for *R. mangle* were used as follows: RmBra20, RmBra45, RmBra50, RmBra59 (Ribeiro et al., 2013) and Rm7, Rm11, Rm19, Rm41, Rm46 (Rosero‐Galindo, Aitan‐Solis, Árdenas‐Henao, Ohme, & Oro‐Perea, 2002). Amplification was performed by PCR. Each 20 μl of the amplification reaction consisted of 10× PCR buffer, 1.5 mM MgCl_2_, 0.2 mM dNTPs, 0.15 μM primer (FW, RV), 1 u of Taqpolimerase, and 2 μl of DNA (50 ng). Amplification was performed on an Applied Biosystems Gene Amp PCR SYSTEM 9,700 thermocycler (Thermo Fisher Scientific, USA) at the following temperatures: initial denaturation of 94°C for 2 min, followed by 37 cycles of 15 s at 94°C, 15 s at 50°C (alignment temperatures for each oligonucleotide were according to Rosero‐Galindo et al., 2002 and Ribeiro et al., 2013), 15 s at 72°C and a final extension of 5 min at 72°C. The PCR products were verified by electrophoresis on 1.5% agarose gels stained with 10 mg/ml ethidium bromide for 40 min at 100 V. PCR products were separated by electrophoresis on denaturing gels of 5% polyacrylamide (19:1 acrylamide bisacrylamide) with 5 M urea and 0.5 X TBE. To the PCR product, 4 μl of a solution of formamide (0.45% bromophenol blue and 0.25% xylene‐cyanol, denatured at 94°C for 5 min) was added. The electrophoresis was performed at 60 W for 1.5 hr in a manual sequencer (SQ3 sequencer, Hoefer Scientific Instruments, San Francisco, CA, USA). The fragments were visualized using the silver staining technique (CIRAD, 2002), using a 10 bp marker as a reference and a white light transiluminator for the fragments reading.

#### 2.3.1 Data analysis

First, to explore how genetic diversity of *R. mangle* is organized in the Yucatan Peninsula, two approaches were used as follows: (a) An individual assignment test was done with a Bayesian approach implemented in the Program STRUCTURE v.2.3.4 (Pritchard, Stephens, & Donnelly, 2000). The program was run with the Admixture model and the LOCPRIOR option (Hubisz, Falush, Stephens, & Pritchard, 2009). A period of burning of 200,000 and 400,000 iterations after the burning period was used. We performed 20 replicates for each *K* value (*K* = 1–15) and checked the consistency of results. The optimal *K* value was calculated using the Δ*K* method described by Evanno, Regnaut, and Oudet (2005) and implemented in the STRUCTURE HARVESTER program (Earl & Von Holdt, 2012). 2). A Principal Coordinates Analysis (PCoA) with mean population genetic distance was performed with the GenAlex v6.5 program (Peakall & Smouse, 2006); this analysis allows the spatial recognition of groups of genotypes, without altering the data and only considers the matrix of genetic similarity. Subsequently, the genetic structure was assessed by Analysis of Molecular Variance (AMOVA) at three hierarchical levels: (a) At regional scale in the Yucatan Peninsula, an analysis was performed for all tall and scrub populations (populations), and one for sites considering tall and scrub individuals within a site indistinctly (sites); (b) for populations and sites between main groups obtained by STRUCTURE and PCoA; (c) at the local scale, tall and scrub populations were analyzed within each site. The statistical significance was tested for all levels from 1,000 permutations in the Arlequin program V. 3.5.2.2 (Excoffier & Lischer, 2010). The Nei's pairwise population genetic distance matrix was calculated with GenAlex V6 program (Peakall & Smouse, 2006). Also, to evaluate the distance isolation hypothesis, Mantel test was done for sites and populations using the geographic distances, obtained as the coast distance between sites (Km) and Nei's genetics distance matrix (Nei, 1978), with 1,000 permutations by the GenAlex V6 program (Peakall & Smouse, 2006).

The genetic diversity of *R. mangle* was evaluated at different organizational levels: Yucatan Peninsula, main groups defined by STRUCTURE and PCoA, sites, populations and by physiognomic type of mangrove within regions (tall and scrub). The evaluated estimators were expected (*H*_E_) and observed heterozygosity (*H*_O_) and allelic richness (*N*a), all estimators were obtained with GenAlex V6 program (Peakall & Smouse, 2006). Comparisons were made with estimators calculated between regions and mangrove types with a validation of 1,000 permutations with the FSTAT program v.2.9.3.2 (Goudet, 2001). Also, inbreeding coefficient (*F*_IS_) was estimated for all populations, regions, and mangrove types, as well as for all the Yucatan Peninsula. The evaluated estimators were compared among populations, regions, and type of mangrove, using 1,000 permutations with FSTAT program ver. 2.9.3.2 (Goudet, 2001).

## 3 RESULTS

### 3.1 Morphologic and environmental variability

**Figure 2:**
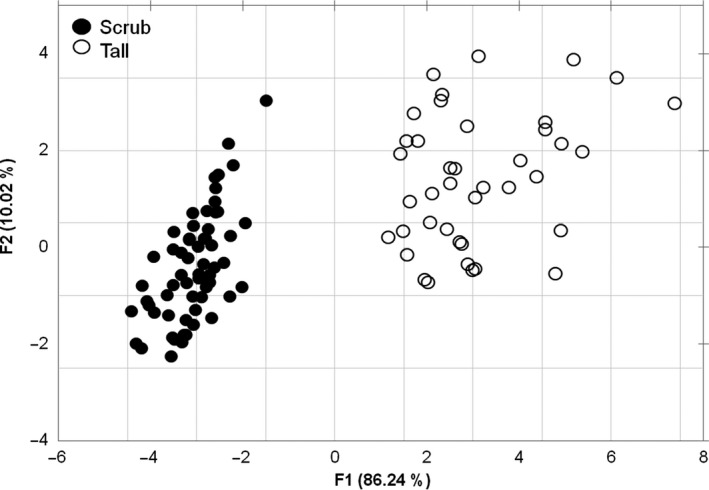
Canonical Discriminant Analysis of the morphologic variables measured in *Rhizophora mangle* of the Yucatan Peninsula

The study populations had high morphological variability within and among sites (Table 1) and, according to the canonical discriminant analysis, they were divided into the preassigned mangrove types: tall and scrub (Figure 2). Tree height was the most discriminant variable (contribute for 86.2% of total morphological variation among populations); it varied from individuals of 0.5 m in Sian Ka'an, to trees of 23 m in Celestún (Table 1). Leaf width and propagule length represented 10% of the total variation (Figure 2; *n* = 130, λ Wilks = 0.009, *p < *0.05).

**Table 1:** Morphologic and environmental variables measured in *Rhizophora mangle* populations from the Yucatan Peninsula, Mexico

Site	Key Pop.	Coordenates	Morphologic variables					Environmental variables		
			Height (m)	Diameter (cm)	Propagule (cm)	Leaf width (cm)	Leaf length (cm)	Salinity (ppt)	PT (mg/g)	PE (g/kg) (%PE)
Celestún	Cel‐T	20.51227°, −90.22377°	16.3 ± 4.8	22.9 ± 7.2	19.4 ± 3.4	6.1 ± 0.4	14.1 ± 1.3	24.5 ± 10	0.99	15.03 (1.5)
	Cel‐S	20.51227°, −90.22377	2.8 ± 1	3.4 ± 1.5	13.7 ± 2.6	4.3 ± 0.5	9.5 ± 0.9	42.7 ± 1.4	1.13	13.62 (1.19)
Mahahual	Mah‐S	18.78512°, −87.74883°	2 ± 0.6	1.8 ± 0.4	11.4 ± 1.8	4.4 ± 0.5	11.2 ± 0.9	0.8 ± 0.12	0.59	1.01 (0.16)
Nichupté	Nic‐T	21.03337°, −86.83741°	6.3 ± 2.7	8.8 ± 1.4	21.8 ± 5.9	5.4 ± 0.8	12 ± 1.7	2.5 ± 0.78	0.62	8.36 (1.33)
	Nic‐S	19.47133°, −87.29121°	1.4 ± 0.3	1.7 ± 0.5	9.3 ± 3.9	3.3 ± 0.3	8.8 ± 1.1	4.7 ± 0.36	0.43	25.06 (5.8)
Progreso	Pro‐T	21.278704°,−89.64454°	8 ± 1.1	8.1 ± 2.2	22.0 ± 4.9	5.1 ± 0.5	11.1 ± 1.2	49 ± 3.75	0.23	12.48 (5.31)
	Pro‐S	21.24614°, −89.83677°	1.5 ± 0.2	2.1 ± 0.4	14.6 ± 3.5	3.8 ± 0.5	9 ± 1.1	122 ± 9.92	0.76	23.35 (3.03)
Puerto Morelos	Pue‐S	20.90477°, −86.85719°	1.5 ± 0.2	1.8 ± 0.3	10.2 ± 2.5	4 ± 0.3	10.7 ± 0.7	9.6 ± 1.53	1.08	7.32 (0.67)
Rio Lagartos	Rio‐T	21.35521°, −88.08343°	9 ± 2.4	5.9 ± 1.8	23.0 ± 2.8	6.0 ± 0.4	12.6 ± 0.8	27.7 ± 2.48	0.35	10.51 (2.95)
	Rio‐S	21.35521°, 88.08349°	1.5 ± 0.4	1.3 ± 0.3	10.6 ± 1.3	4.1 ± 0.6	10.2 ± 1.1	9.4 ± 3.4	0.53	5.81 (1.09)
Sian Ka'an	Sia‐T	19.7800°, −87.4789°	14 ± 2.4	39 ± 2.9	24.3 ± 4.8	6 ± 0.5	12.5 ± 1.2	30.3 ± 1.7	0.55	18.61 (3.34)
	Sia‐S1	19.80683°, −87.53764°	0.45 ± 0.1	1.7 ± 0.4	11.5 ± 2.2	4.2 ± 0.6	9.6 ± 0.9	29.9 ± 3.3	0.84	6.65 (0.79)
	Sia‐S2	19.82301°, −87.49882°	1 ± 0.3	1.3 ± 0.3	16.8 ± 3.5	4.5 ± 0.8	9.8 ± 1.2	49.6 ± 2.3	0.25	12 (4.84)

Note

For key populations: T = tall and S = scrub; diameter (measured 20 cm above the last aerial root); PT: Total Phosphorus; PE: Extractable phosphorus (% of extractable Phosphorus, PE/PT*100).

**Figure 3:**
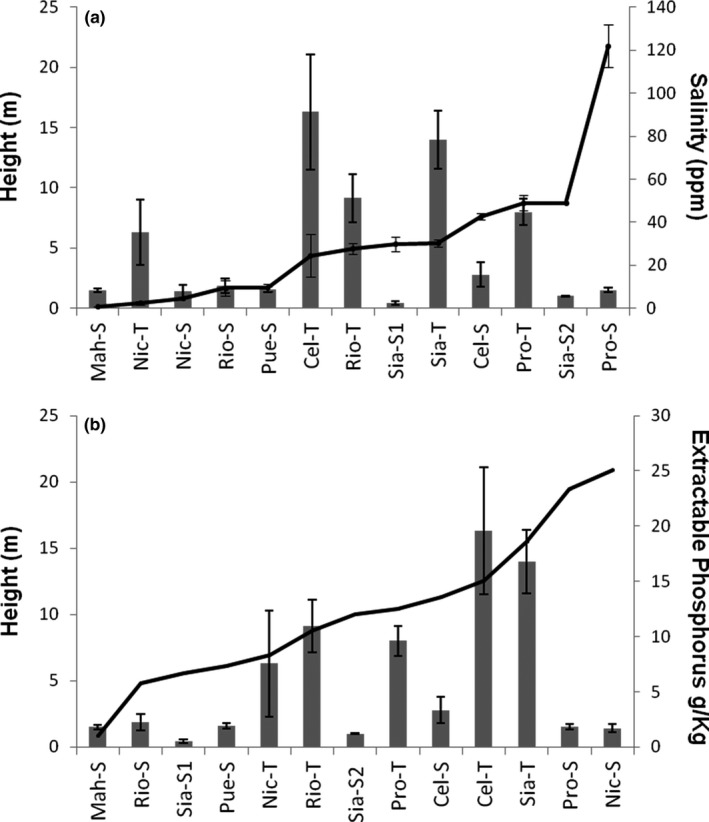
Relationship among height, salinity, and extractable phosphorus for 13 populations of *Rhizophora mangle* in the Yucatan Peninsula (see Table 1 for abbreviations). Bars are mean height ± standard deviation; lines are mean ± standard deviation of (a) interstitial salinity and (b) extractable phosphorus

**Figure 4:**
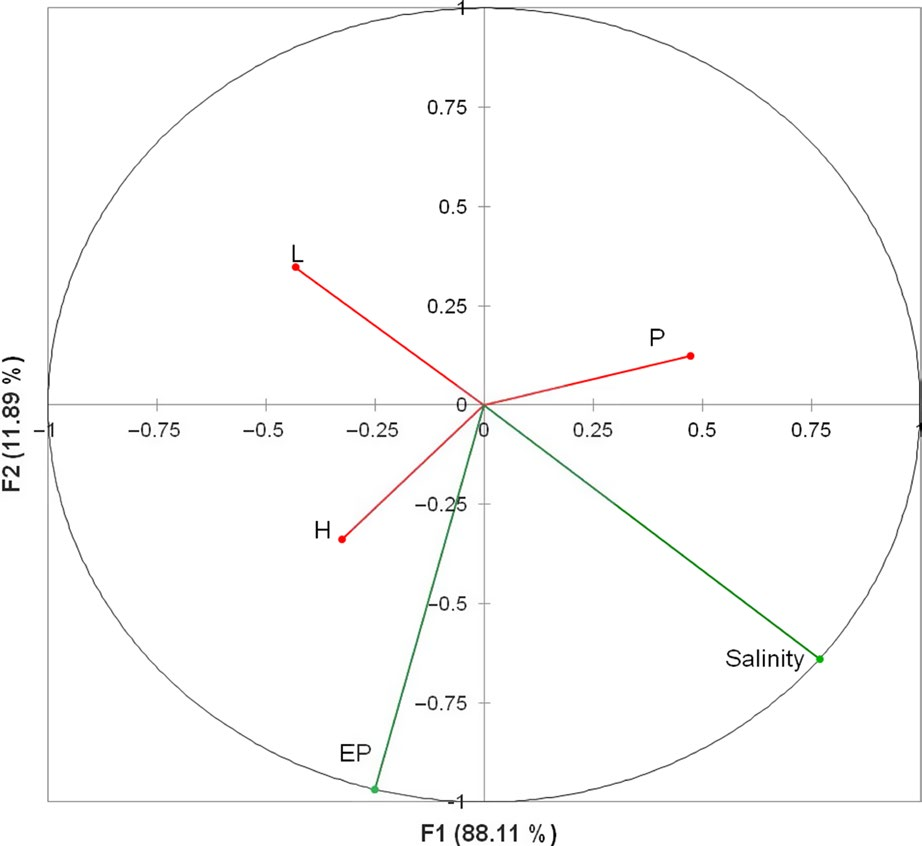
Canonical Correlation Analysis of morphological (H: height of tree, L: leaf area, P: propagule length) and environmental variables (*n* = 130, Canonical correlation = 0.36, λ Wilks = 0.85, *p < *0.05)

Interstitial salinity varied from 0.8 ppt in scrub‐Mahahual to 122 ppt in scrub‐Progreso (Table 1). The population with the highest total phosphorus (P) content was scrub‐Celestún (1.13 mg/g) and the lowest total P content was in tall‐Progreso (0.23 mg/g). The extractable P was the highest in scrub‐Nichupté (25.06 mg/kg) and the lowest in scrub‐Mahahual (1.01 mg/kg). The percentage of extractable P in the sites was less than 6% in all cases (Table 1), and no direct relationship was found between tree height and salinity or extractable P (EP; Figure 3a,b). However, the relationship between environmental and morphological variables was supported by the Canonical Correlation Analysis. The first factor explained 92.45% of the total variation, and a significant correlation among morphology variability, salinity, and EP was also observed (0.36, *p* < 0.05). However, the λ Wilks showed a poor predictive power (0.85). This analysis also showed that salinity was the variable that contributed the most to the variability in morphology and had a negative correlation with tree height and leaf area, but EP had a positive correlation with these latter variables (Figure 4).

**Figure 5:**
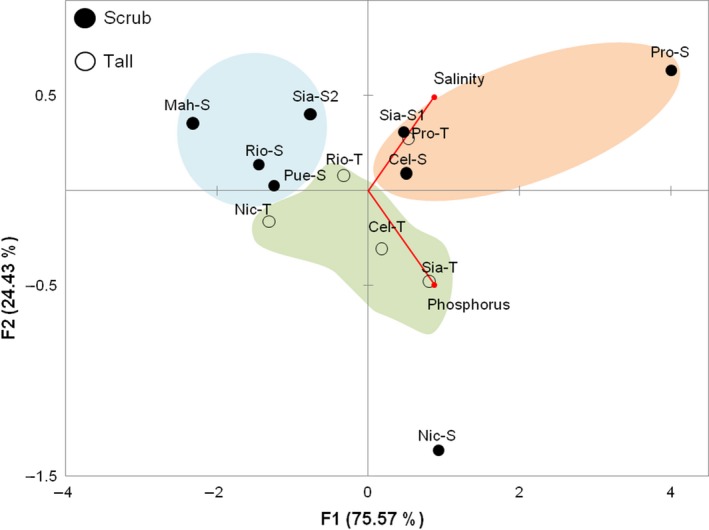
Principal Component Analysis of interstitial salinity and extractable phosphorus (measured at 30 cm depth) in 13 *Rhizophora mangle* populations (see Table 1 for abbreviations)

The response of the two physiognomic types of mangrove to salinity and EP correlation was supported by the Principal Component Analysis; we found that: Sites with high salinity and low or high EP had scrub mangroves (Sia‐S1, Cel‐S, Pro‐S); sites with low salinity and high EP values had tall mangroves (Sia‐T, Cel‐T, Rio‐T, Nic‐T); and sites with low salinity and EP values had scrub mangrove (Mah‐S, Rio‐S, Pue‐S, Sia‐S2) (Figure 5). Two sites did not respond to these trends: scrub‐Nichupté with the highest values of EP and low salinity, and tall‐Progreso with high salinity and low EP values.

### 3.2 Genetic variability

**Figure 6:**
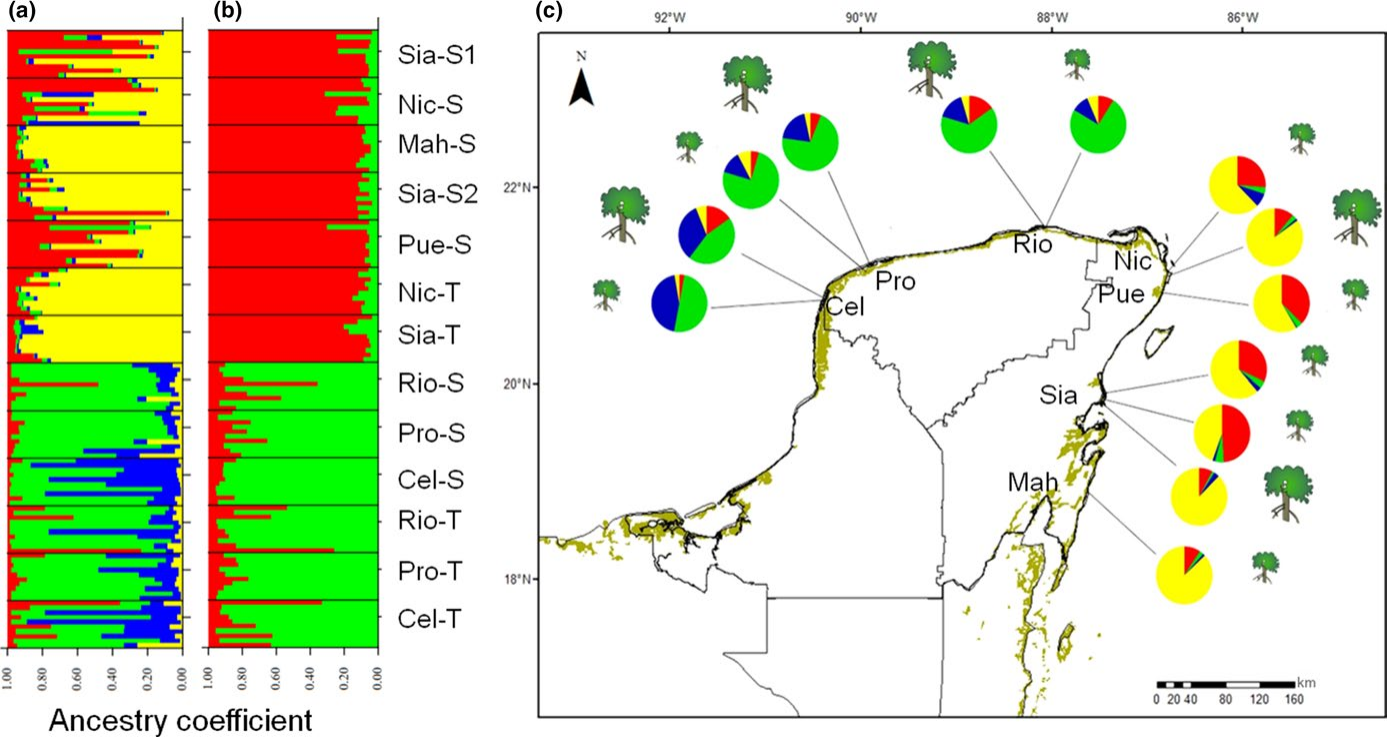
Bayesian assignment analysis performed in STRUCTURE for 130 individuals of *Rhizophora mangle*. (a) *K* = 4 (Δ*K* = 4.79), substructure within each region (Gulf of Mexico: blue‐green, Caribbean Sea: red‐yellow). (b) *K* = 2 (Δ*K* = 4.38), correspond to the oceanographic regions: Gulf of Mexico (green) and Caribbean Sea (red). (c) The map represents the Yucatan Peninsula and the populations of *Rhizophora mangle* sampled (see Table 1 for abbreviations), the pie charts represent Q value for *K* = 4

The 130 individuals of *R. mangle* sampled in the Yucatan Peninsula were divided into four groups according to the optimal *K* obtained with the Evanno method (*K* = 4, Δ*K* = 4.79). Moreover, the STRUCTURE analysis based in *K* = 4 presented two main groups: one group composed of the populations from the Gulf of Mexico coast: Celestún (Cel‐T and Cel‐S), Rio Lagartos (Rio‐S, Rio‐T), and Progreso (Pro‐T, Pro‐S); and another group integrated by the populations from the Caribbean coast: Nichupté (Nic‐S), Puerto Morelos (Pue‐S), Sian Ka'an (Sia‐S1, Sia‐S2, Sia‐T), and Mahahual (Mah‐S) (Figure 6a). Also, two subgroups were detected within each of these groups, which explains the value of *K* = 4 thrown by the Evanno's method. However, the admixed individuals, because of the share ancestry of the populations, hindered the identification of clearly identified subgroups (Figure 6a,c). Although considering different values of *K* may reflect different genetic and demographic processes, and ensure a better biological interpretation of the data (Meirmans, 2015), the second high ΔK obtained by the Evanno's method was also considered (*K* = 2, Δ*K* = 4.38). This reinforces the existence of the two main groups, the Gulf of Mexico and the Caribbean Sea (Figure 6b). Because both coasts belong to different oceanographic regions, henceforth we will refer those groups as regions.

**Figure 7:**
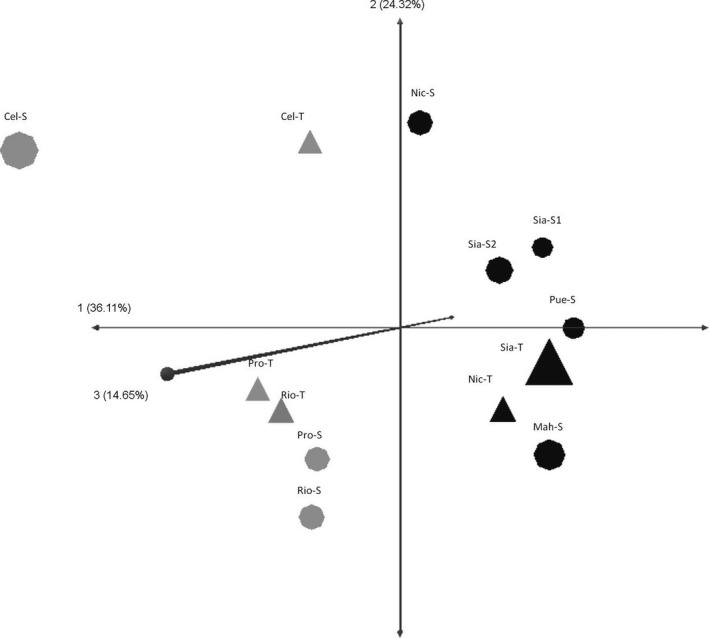
Principal Coordinate Analysis in three dimensions, performed withnine microsatellite loci and 13 populations of *Rhizophora mangle*. Axis 1 (36.11%) divides the Gulf of Mexico region and the Caribbean Sea; Axes 2 and 3 (38.97%) indicate the genetic difference within each region. Populations of the Caribbean Sea are indicated as closed black symbols and populations of the Gulf of Mexico as closed gray symbols; circles represent tall populations and triangles scrub populations (see Table 1 for abbreviations)

The grouping pattern obtained with STRUCTURE was partially supported by the three‐dimensional Principal Coordinate Analysis (PCoA; Figure 7). Coordinate 1 explained the 36.11% of the variation and reinforced the existence of the two genetically different regions (Gulf of Mexico and Caribbean Sea); coordinates 2 and 3 explained the 38.99% of the variation and placed scrub and tall populations from the same site farther away than populations from different sites. The Nei's genetic distance matrix reinforces the distance observed on PCoA (Table 2). The tall and scrub populations within a site (with distances of few kilometers) had equal genetic distances, or even higher, than distances between populations from different sites. For instance, the genetic distance between Cel‐S and Cel‐T (0.12) was the same distance than that between Cel‐S and Rio‐S (0.12); or the genetic distance between Sia‐T and Sia‐S2 (0.2), which was higher than the distance between Sia‐T and Pue‐S (0.17; Table 2).

**Table 2:** Nei's (1978) pairwise genetic distances between populations of *Rhizophora mangle* in the Yucatán Peninsula

Pob	Cel‐S	Cel‐T	Pro‐T	Pro‐S	Rio‐S	Rio‐T	Sia‐T	Sia‐S1	Mah‐S	Nic‐S	Pue‐S	Sia‐S
Cel‐T	0.12											
Pro‐T	0.13	0.09										
Pro‐S	0.14	0.11	0.06									
Rio‐S	0.12	0.13	0.05	0.06								
Rio‐T	0.12	0.09	0.08	0.07	0.05							
Sia‐T	0.16	0.22	0.24	0.18	0.17	0.20						
Sia‐S1	0.17	0.11	0.13	0.10	0.11	0.13	0.08					
Mah‐S	0.20	0.17	0.15	0.13	0.09	0.14	0.06	0.06				
Nic‐S	0.13	0.07	0.12	0.14	0.14	0.14	0.17	0.10	0.11			
Pue‐S	0.27	0.19	0.19	0.15	0.11	0.18	0.17	0.09	0.10	0.11		
Sia‐S2	0.29	0.12	0.16	0.13	0.15	0.17	0.20	0.06	0.12	0.11	0.05	
Nic‐T	0.25	0.16	0.19	0.13	0.14	0.12	0.16	0.14	0.07	0.11	0.15	0.15

The genetic structure of *R. mangle* in the Yucatan Peninsula showed by the AMOVA indicated that the studied populations differed 13% (*p < *0.001). Also, the genetic differentiation between regions was 6.5% (*p < *0.001, Table 3). The analysis performed by sites (including indistinctly tall and scrub individuals within sites) showed a lower genetic differentiation (9%, *p < *0.05) than by populations. However, the differentiation between regions was similar (6%, *p < *0.05) (Table 3). At local scale, the genetic differentiation of tall and scrub populations within sites was high and for some sites even higher than for all the Yucatan Peninsula, with values from 3% to 13.11% (Table 4). A larger genetic differentiation was observed in a site of the Caribbean Sea coast, between tall and scrub populations of Sian Ka'an (13.11%, *p < *0.05). The lowest genetic differentiation was observed among tall and scrub populations of Rio Lagartos (3%, *p > *0.05). With exception of Celestún, sites from the Gulf of Mexico had less genetic differentiation between physiognomic types than those from the Caribbean Sea (Table 4). The relation among geographic distance and Nei's genetic distance demonstrated by Mantel test among sites of the Yucatan Peninsula showed a significant relation (*R* ^2^ = 0.52, *p < *0.001); while the analysis made by populations presented a weak but significant relation (*R* ^2^ = 0.21, *p < *0.001). Within regions, the Mantel test did not present significant relationship.

**Table 3:** AMOVA's performed for *Rhizophora mangle* at regional scale in the Yucatan Peninsula, for all populations/sites (a) and between populations/sites of Caribbean Sea and Gulf of México regions (b)

Source of variation	*df*	Region scale			
		Sum of squares	Variance components	Population % variation	Site % variation
(a) Yucatan Peninsula					
Among populations	12	84.01	0.26	13.11**	9.01*
Within populations	247	430.5	1.74	86.89	90.99
(b) Caribbean Sea/Gulf of Mexico					
Among regions	1	22.82	0.13	6.46**	5.64*
Among populations within regions	11	61.199	0.19	9.24**	5.46**
Within populations	247	430.5	1.74	84.3**	88.8**

a *p *< 0.05.

b *p* < 0.001.

**Table 4:** AMOVAs performed at local scale for scrub and tall populations of *Rhizophora mangle* within sites of Caribbean Sea (a, b) and Gulf of México (c,d,e) in the Yucatan Peninsula

Local scale									
Caribbean Sea					Gulf of Mexico				
Source of variation	*df*	Sum of squares	Variance components	% variation	Source of variation	*df*	Sum of squares	Variance components	% variation
a) Sian ka'an	1	9.167	0.275	13.11**	c) Celestún	1	6.600	0.24	11.79*
Within populations	58	105.85	1.825	86.89	Within populations	38	68.250	1.796	88.2
b) Nichupté	1	6.025	0.207	9.88**	d) Progreso	1	3.250	0.076	4.19*
Within populations	38	71.7	1.887	90.12	Within populations	38	65.900	1.734	95.81
					e) Rio Lagartos	1	3.150	0.061	3.08
					Within populations	38	73.100	1.924	96.92

a *p *< 0.05.

b *p* < 0.001^.^

### 3.3 Genetic diversity

The nine microsatelite loci used detected a total of 35 alleles. With the exception of the RM46 locus, all loci were polymorphic. The Yucatan Peninsula had an expected (*H*_E_) and observed heterozygosity (*H*_O_) of 0.37 ± 0.003 and 0.27 ± 0.003, respectively, and an allelic richness (*N*a) of 2.41 ± 0.02. Also, the *H*_O_ was higher for the Caribbean Sea region (0.37 ± 0.01) than for the Gulf of Mexico region (0.23 ± 0.02), but their differences between *H*_E_ and *H*_O_ were lower (Table 5). The *N*a was similar in both regions, 2.38 ± 0.05 in the Gulf of Mexico and 2.49 ± 0.08 in the Caribbean Sea (Table 5). Concerning mangrove types, the tall populations showed the greatest genetic diversity in the Gulf of Mexico region (*N*a = 2.52 ± 0.08, *H*_O_ = 0.26 ± 0.02), while the scrub populations showed the highest values of genetic diversity in the Caribbean Sea region (*N*a = 2.53 ± 0.09, *H*_O_ = 0.3 ± 0.01). The population with the highest *H*_O_ was tall‐Nichupté while scrub‐Celestún had the lowest *H*_O;_ *N*a varied from 1.89 for tall‐Sian Ka'an to 2.77 from tall‐Rio Lagartos, scrub‐Nichupté and scrub‐Sianka'an1. The *H*_E_ was higher than *H*_O_ in all populations; however, the populations in the Gulf of Mexico had the highest difference, especially in the site of Celestún (Table 5). The comparison between regions and mangrove types and among populations did not show significant differences, using the different estimators evaluated.

**Table 5:** Genetic diversity estimators for sites/populations sampled of *Rhizophora mangle* in the Yucatan Peninsula ±95% confidence intervals

Region	Site	Physiognomic type of mangrove	Na	*H*_E_	*H*_O_	*F*_IS_
Gulf of Mexico			**2.38 ± 0.05**	**0.37 ± 0.01**	**0.23 ± 0.12**	**0.35 ± 0.02**
	Celestún		2.67 ± 0.14	0.39 ± 0.04	0.19 ± 0.04	0.48 ± 0.05
		T	2.44 ± 0.15	0.41 ± 0.04	0.24 ± 0.04	0.38 ± 0.08
		S	2.22 ± 0.02	0.31 ± 0.06	0.14 ± 0.04	0.48 ± 0.08
	Progreso		2.25 ± 0.16	0.38 ± 0.04	0.25 ± 0.03	0.29 ± 0.08
		T	2.33 ± 0.20	0.35 ± 0.04	0.22 ± 0.02	0.27 ± 0.05
		S	2.22 ± 0.17	0.38 ± 0.04	0.28 ± 0.06	0.26 ± 0.10
	Rio Lagartos		3.00 ± 0.19	0.41 ± 0.03	0.25 ± 0.02	0.37 ± 0.04
		T	2.77 ± 0.22	0.46 ± 0.04	0.32 ± 0.04	0.29 ± 0.06
		S	2.33 ± 0.17	0.33 ± 0.04	0.18 ± 0.04	0.42 ± 0.11
	Totals	T	2.52 ± 0.08	0.40 ± 0.02	0.26 ± 0.02	0.32 ± 0.02
		S	2.26 ± 0.02	0.34 ± 0.01	0.20 ± 0.03	0.39 ± 0.04
Caribbean Sea			**2.49 ± 0.08**	**0.37 ± 0.01**	**0.31 ± 0.01**	**0.19 ± 0.02**
	Mahahual	S	2.00 ± 0.08	0.35 ± 0.03	0.29 ± 0.04	0.25 ± 0.06
	Nichupté		3.00 ± 0.18	0.42 ± 0.03	0.33 ± 0.03	0.25 ± 0.04
		T	2.44 ± 0.18	0.38 ± 0.03	0.36 ± 0.03	0.06 ± 0.02
		S	2.77 ± 0.20	0.39 ± 0.03	0.30 ± 0.03	0.19 ± 0.04
	Puerto Morelos	S	2.66 ± 0.20	0.37 ± 0.03	0.31 ± 0.04	0.29 ± 0.06
	Sian Ka'an		2.36 ± 0.14	0.42 ± 0.03	0.29 ± 0.03	0.29 ± 0.03
		T	1.89 ± 0.15	0.31 ± 0.02	0.26 ± 0.01	0.09 ± 0.03
	1	S	2.77 ± 0.25	0.43 ± 0.03	0.33 ± 0.03	0.22 ± 0.05
	2	S	2.44 ± 0.23	0.39 ± 0.04	0.28 ± 0.03	0.26 ± 0.05
	Totals	T	2.17 ± 0.18	0.34 ± 0.02	0.31 ± 0.03	0.08 ± 0.01
		S	2.53 ± 0.09	0.39 ± 0.11	0.30 ± 0.01	0.24 ± 0.01
Yucatan Peninsula			**2.41 ± 0.02**	**0.37 ± 0.003**	**0.27 ± 0.003**	**0.26 ± 0.01**

Note

*N*a: Allelic richness; *H*_E_: expected heterozygosity; *H*_O_: observed heterozygosity; *F*_IS_: Inbreeding coefficient; Type of mangroves: Tall, T and Scrub, S.

Bold values indicate averages for the regions and for all the Yucatan Península.

The inbreeding coefficient (*F*_IS_) for the populations showed positive significantly values in all populations (from 0.06 ± 0.02 in Nic‐T to 0.48 ± 0.05 in Cel‐S). The *F*_IS_ for the Yucatan Peninsula was 0.26 ± 0.01. The Caribbean Sea region had a significantly lower *F*_IS_ (*p < *0.05) than that of the Gulf of Mexico (0.19 ± 0.02 and 0.35 ± 0.02, respectively; Table 5). Tall populations had lower *F*_IS_ (0.20 ± 0.03) than scrub populations (0.31 ± 0.02), but values were not significantly different.

## 4 DISCUSSION

### 4.1 Morphologic variability

Although salinity and phosphorous (P) are two of the main factors that have been related to mangrove forest structure, especially in scrub mangroves (Feller et al., 2003; Lin & Stenberg, 1992a; Lovelock et al., 2006, 2004 ; Naidoo, 2006, 2010 ), in our study, no direct relation of these variables with height was observed (Figure 3a,b). Instead, the mangrove height responded partially to the interaction of the effects of salinity and P (Figure 4). This agrees with the relationship observed among P assimilation and water availability in mangroves (Mckee, Feller, Popp, & Wanek, 2002; Medina, Cuevas, & Lugo, 2010). As salinity increases and water availability diminishes, P assimilation can also decrease with consequent growth reduction (Naidoo, 1987).

Nevertheless, given the low correlation and poor predictability of the environmental variables, other variables can interact in the synergy that controls morphological structure. One of them can be the hydroperiod, which regulates the resources and modifies stress factors of a site (Feller et al., 2010; Lin & Sternberg, 2007; Twilley & Rivera‐Monroy, 2005). Then, the higher P values for scrub‐Nichupté could presumably be for changes in the hydrological dynamics caused by road construction, and the resulting wastewater discharges (Vázquez‐Lule, Santos‐González, & Adame, 2009).

High phenotypic plasticity is related to species adapted to heterogeneous environments and it has important ecological and evolutionary implications (Crispo, 2008; Gianoli, 2004). Mangroves are characterized by a high heterogeneous landscape with environmental settings characterized by high stress factors that control structure and function of populations (Feller et al., 2010; Twilley & Rivera‐Monroy, 2005). Similarly, other aquatic species also show a great phenotypic plasticity at very fine scales (Kudoh & Whigham, 1997; Millet, Kristjánsson, Einarsson, & Räsänen, 2013). This plasticity, resulting from the environmental variability, can be an important factor in evolutionary diversification, because it plays a significant role in the relationships among divergent selection, adaptive divergence, and gene flow (Crispo, 2008; Nosil et al., 2009).

### 4.2 Regional genetic variability

The genetic diversity of *R. mangle* in the Yucatan Peninsula (*H*_O_ = 0.27 ± 0.02) was greater than that reported for the Baja California Peninsula in Mexico (*H*_O_ = 0.16, Sandoval‐Castro et al., 2012) and even higher than that reported for all Mexico (*H*_O_ = 0.25 ± 0.03, Sandoval‐Castro et al., 2014). These results can be attributable to the two physiognomic types that we considered per site, which involves ecological differences between samples within a site. Studies on insects, birds, mammals, and mollusks have shown that sampling different ecotypes result in different levels of genetic diversity (Fruet et al., 2017; Lu, Wang, Li, & Liu, 2016; Nosil, 2007; Spurgin, Illera, Jorgensen, Dawson, & Richardson, 2014). Additionally, for conservation biology, it is important to consider possible sources of greater genetic diversity because this represents relevant variability for species persistence and their evolutionary potential (Piñero et al., 2008). For mangroves, for instance, Dahdouh‐Guebas et al. (2004) have observed significantly different allele frequencies between seaward and landward of the gray mangrove *Avicennia marina*. Moreover, although our study considered fewer individuals per population than other studies, our results for similar sites were comparable. Sandoval‐Castro et al. (2014) reported values of genetic diversity for Progreso (*H*_E_ = 0.36, *H*_O_ = 0.29) and Puerto Morelos (*H*_E_ = 0.38, *H*_O_ = 0.31), which are similar to our values for the same sites (Progreso *H_E_* = 0.35 ± 0.08, *H_O_* = 0.22 ± 0.07; Puerto Morelos *H_E_* = 0.37 ± 0.08, *H_O_* = 0.31 ± 0.11). Future studies should include more individuals per physiognomic type of mangrove within a site to validate the sample size.

In addition, our study showed, for the first time, that the genetic diversity of *R. mangle* in the Yucatan Peninsula is clearly arranged in two regions: the Gulf of Mexico and the Caribbean Sea coasts (Figure 6a, c), which correspond to two oceanographic regions (Wilkinson et al., 2009). Based on other molecular markers, differences on allele frequency among these regions have also been observed for populations of *R. mangle* (Núñez‐Farfán et al., 2002) and *Avicennia germinans* (Nettel & Dodd, 2007) and this differentiation can be explained by the geomorphologic history of the peninsula. The relief and karst formation of the Yucatan Peninsula consists in two main stages: an ancient one of the Miocene‐Pliocene in the south and east on the Caribbean Sea, and another essentially of the Pleistocene in the north and Gulf of Mexico (López‐Ramos, 1973; Lugo‐Hubp, Aceves‐Quesada, & Espinosa‐Pereña, 1992). During the earliest stage of the peninsula, fossil records locate *R. mangle* in Mexico and South America (Graham, 2006; Langenheim, Hackner, & Bartlett, 1967; Tomasini‐Ortiz & Martínez‐Hernández, 1984). After the last Pleistocene glaciations, in the Holocene, temperatures began to rise gradually and *R. mangle* expanded even further beyond the tropical belt (Gutiérrez‐Ayala, Torrescano‐Valle, & Islebe, 2012; Pil et al., 2011; Sandoval‐Castro et al., 2014). The lower genetic diversity, the larger difference between *H*_O_ and *H*_E_ and the higher *F*_IS_ for populations in sites of the Gulf of Mexico than in populations of the Caribbean Sea found in this study suggest a founder effect due to a colonization of *R. mangle* during the Holocene.

At the most recent glacial period, an extreme geographic isolation took place for many species, with subsequent species‐specific patterns of postglacial expansion (Kennedy et al., 2016). In mangroves, the influence on genetic variability attributable to postglacial establishment has been reported in America for *R. mangle* and *A. germinans* (Cerón‐Souza et al., 2015; Pil et al., 2011). Ocean currents are another factor that plays a role in the genetic structure of the hydrochoerus species *R. mangle* (Cerón‐Souza et al., 2015; Pil et al., 2011). In Brazil, the northern and southern populations of *R. mangle* have a strong relationship between geographic and genetic distance (*R* ^2^ = 0.5) due to currents that interrupt continuous genetic flow along the Brazilian coast (Pil et al., 2011). In the Yucatan Peninsula, a similar relationship (*R* ^2^ = 0.52, *p* < 0.05) was found; the upwelling of deep waters at Cabo Catoche in the northeastern of the Peninsula interrupts the continuous flow of the Yucatan current, which drives to northeast Gulf of México as the loop current (Figure 1b; Laurindo, Mariano, & Lumpkin, 2017). This current disruption prevents the continuous dispersal of *R. mangle* propagules along the coast from the Caribbean Sea to the Gulf of Mexico, preventing the genetic flow and keeping genetic differentiation between both regions (Martínez & Pares, 1998; Wilkinson et al., 2009).

In our study, no morphological differences between regions were found, which reflect that the genetic variability did not respond to historical differences between populations, but to local adaptation to environment. For instance, European oaks do not show any association between genetic divergence connected to colonization events and those associated with local selection pressures (Kremer et al., 2002). Therefore, it is important to consider different scales, which can include local adaptation traits to environment and reveal the colonization history of populations. Several factors regulate population genetic structure in natural landscapes and also provide insights into the complex interactions between the environment and the genome that influence the distribution of species, and mediate phenotypic adaptation to local conditions (Bragg, Megan, Andrew, & Justin, 2015; Orsini et al., 2013).

### 4.3 Local genetic variability

Tall and scrub populations of *R. mangle* showed significant genetic differences that could be caused by their contrasting environments within a site (Table 1). These environmental differences could represent a reproductive barrier, because of the influence of salinity and nutrient availability on autogamy rate, flowering season, fruit production and ripening, and size of propagules (Coupland, Paling, & McGuiness, 2006; Klekowski, Lowenfeld, & Hepler, 1994; Lowenfeld & Klekowski, 1992; Proffit & Travis, 2010; Sánchez‐Núñez & Mancera‐Pineda, 2011). Thus, the genetic distance between tall and scrub populations within a site could be explained, in part, for the high autogamy rate characteristic of the species and also to an asynchronous phenology between adjacent populations that interrupts gene flow (Sánchez‐Núñez & Mancera‐Pineda, 2011). On the other hand, the intricate root system of *R. mangle*, especially in the scrub mangrove, represents a physical barrier for propagules dispersion (Tonné, Beeckman, Robert, & Koedam, 2017; Van der Stocken & Menemenlis, 2017; Van der Stocken et al., 2015). This suggests that scrub populations have more limited dispersion, which promotes genetic differentiation within a site (Table 4). Also, in scrub mangroves, the smaller size of propagules indicates fewer stored reserves, growth rate and resistance to wind and wave force that could produce a negative selection against them (Boizard & Mitchell, 2011; Dissanayake et al., 2014; Farnsworth & Ellison, 1996; Huxham et al., 2010; Lin & Sternberg, 1995; Nosil, Vines, & Funk, 2005; Proffit & Travis, 2010; Tomlinson, 1986).

The environmental and ecological differences between tall and scrub mangroves, which coexist at short distances, could be generating breeding barriers that limit the genetic flow between them and promoting a greater genetic differentiation even more than among sites (Tables 2 and 4). The higher *F*_IS_ found in the scrub than in the tall mangrove populations in the Caribbean Sea indicate that the autogamous nature of the *R. mangle* has accentuated the differences between populations at local scale by genetic drift. The main adaptive advantage of gene drift, despite the low diversity associated with inbreeding, denotes reproductive safety and adaptation to a particular niche (Loveless & Hamrick, 1984). Genetic differentiation is a consequence of the combined effects of natural selection and gene drift, which could fix certain genotypes linked to environmental variables that result in greater adequacy, and whose effect is counteracted with the gene flow (Bragg et al., 2015; De Kort et al., 2014). Although this work was performed with neutral markers, natural selection at a locus can affect the frequency of alleles in the loci attached to them due to the *hitch‐hiking* effect (Smith & Haigh, 1974). Moreover, the morphological plasticity responds to environmental variables, but it may also be under genetic control and respond to pressures of natural selection; thus, genotypes with greater morphological and functional plasticity will be more advantageous in wide environmental ranges (Crispo, 2008; Gianoli, 2004).

The genetic variability at local scale driven by environmental variables has also been reported before for other species (DeWoody et al., 2015; Mosca, González‐Martínez, & Neale, 2014; Parisod & Christin, 2008). For mangrove species, genetic differences for *Avicennia* species between populations at a fine scale have been found using RAPDs markers and microsatellites (Dahdouh‐Guebas et al., 2004; Mori, Zucchi, & Souza, 2015). Lira‐Medeiros et al. (2010) reported epigenetic differences in morphologically contrasting populations (1.9–7.5 m) of *Laguncularia racemosa.* Also, genetic differences using AFLP markers were reported among salt marsh and riverside *A. schaueriana* (Lira‐Medeiros, Cardoso, Fernandes, & Gomes‐Ferreira, 2015). Nevertheless, to date, no studies have considered the physiognomic type of *R. mangle* mangrove, environmental variables, or ecological characteristics of our study. Generally, the majority of population genetic studies focus on the classic pattern of isolation by distance, and studies on mangroves are not the exception; however, the idea that adaptive responses to divergent natural selection may impact genomewide population structure has gained momentum and needs to be considered in future studies (Nosil et al., 2009; Orsini et al., 2013).

The genetic structure of *R. mangle*, observed at local scale in the Yucatan Peninsula, suggests that each site is probably composed of demes, where the ecological limits could be generating reproductive barriers. In continuous populations, recurrent processes, such as gene flow, genetic drift, and selection, act in concert to shape the genetic structure (Latta, 2003; Lenormand, 2002). Fine‐scale genetic differentiation has often been reported in other plant populations, mammals, fishes, and insects (Dewoody et al., 2015; Fruet et al., 2017; Nosil, 2007; Vekemans & Hardy, 2004), even under substantial gene flow, suggesting that strong selective pressure promotes local adaptation at small scale in heterogeneous landscapes (Linhart & Grant, 1996; Parisod & Christin, 2008).

The two different types of *R. mangle* considered in our study could probably allow us to find a greater genetic structure in the Yucatan Peninsula in comparison with the reported studies that include a greater number of populations distributed in larger geographic areas (Cerón‐Souza et al., 2015; Sandoval‐Castro et al., 2014). Also, our study showed higher levels of genetic diversity in the Yucatan Peninsula using fewer individuals by population than other studies; so, we suggest that sampling should consider ecological differences between populations or fine genetic structure within a site (Dahdouh‐Guebas et al., 2004; Mori et al., 2015). This approach is important for conservation strategies, for adaptation to future potential environmental changes, and to elucidate the possible natural divergent selection that can be acting in populations with contrasting morphologies at local scales. This would help to the understanding of the processes involved in adaptive selection, and also to distinguish the related environmental variables (Arnaud‐Haond et al., 2006; Garnier‐Géré & Ades, 2001). Further studies need to explore morphological characteristics as adaptive, heritable characters and those that are the result of phenotypic plasticity. This research opens the door to a more comprehensive analysis of ecological considerations in the study of mangrove genetic variability.

## AUTHORS’ CONTRIBUTIONS

D.J.C.delaC., J.M.C., J.H.S., and J.L.A. conceived and designed the study; D.J.C.delaC., M.O.G., and R.U.S. collected the data; D.J.C.delaC., L.Y.E., and J.M.C. analyzed the data; D.J.C.delaC, J.M.C., and J.L.A. wrote the text; all authors provided editorial advice and approved the final version.

## DATA ACCESSIBILITY

Morphological and environmental data and microsatellite genotypes are available at https://doi.org/10.5061/dryad.1578ks0.
